# Implementation of Pharmacogenomic Information on Stevens-Johnson Syndrome and Toxic Epidermal Necrolysis

**DOI:** 10.3389/fmed.2021.644154

**Published:** 2021-03-24

**Authors:** Eri Tsukagoshi, Yoichi Tanaka, Yoshiro Saito

**Affiliations:** Division of Medicinal Safety Science, National Institute of Health Sciences, Kawasaki, Japan

**Keywords:** Stevens-Johnson syndrome, toxic epidermal necrolysis, pharmacogenomics, implementation, drug label, guideline

## Abstract

Drug-related Stevens-Johnson syndrome and toxic epidermal necrolysis (SJS/TEN) are rare but severe adverse drug reactions, termed as idiosyncratic reactions; however, predicting their onset remains challenging. Pharmacogenomic information associated with SJS/TEN has accumulated on several drugs in the last 15 years, with clinically useful information now included on drug labels in several countries/regions or guidelines of the Clinical Pharmacogenetics Implementation Consortium (CPIC) for implementation. However, label information might be different among countries. This mini-review summarizes pharmacogenomic information on drug labels of five drugs in six countries and compared descriptions of drug labels and CPIC guidelines. Finally, we discuss future perspectives of this issue. Pharmacogenomic information on drug labels is not well-harmonized across countries/regions, but CPIC guidelines are a scientifically sound goal for future pharmacogenomic implementation.

## Introduction

Stevens-Johnson syndrome (SJS) and toxic epidermal necrolysis (TEN) are rare but life-threatening, severe adverse drug reactions. SJS and TEN are characterized as fever and mucosal disorders (such as at the mouth and ocular conjunctiva) and are reactions within the same spectrum (1, 2). Skin lesions often exhibit epidermal necrosis, resulting in skin detachment such as erosion and blisters. Top 5 common causative drugs were reported to be carbamazepine, allopurinol, phenytoin, lamotrigine and sulfamethoxazole in Asians and allopurinol, carbamazepine, sulfamethoxazole, phenytoin and phenobarbital in Europeans (3). In Japan, diagnostic classification is defined as follows: SJS; skin detachment area <10% of the body surface area; TEN, not <10% of the body surface area (4), with the associated mortality estimated at 1–5% and 20–30%, respectively (5, 6). Hence, they are considered the most important severe adverse reactions from a pharmacovigilance standpoint and in terms of patient relief. However, they are called idiosyncratic reactions, and predicting their onset has remained challenging.

In 2004, Chung et al. reported a markedly strong association between HLA-B^*^15:02 and carbamazepine-related SJS (7). To date, pharmacogenomic information associated with SJS/TEN has accumulated on several drugs. Accordingly, clinically useful pharmacogenomic information, as well as and their application, are included on drug labels in several countries/regions or guidelines of the Clinical Pharmacogenetics Implementation Consortium (CPIC). However, label information may differ among countries. This mini-review summarizes the pharmacogenomic information on drug labels of allopurinol, carbamazepine, oxcarbazepine, phenytoin, and fosphenytoin in six countries/regions.

## Allopurinol

Allopurinol is used as a urate-lowering drug and frequently causes severe cutaneous adverse reactions (SCARs), including SJS/TEN (8–10). The frequency of allopurinol-related SCARs is estimated to be 0.1–0.4%. The HLA-B^*^58:01 allele is a strong predictor for allopurinol-related SCARs and the population/ethnic frequency of this *HLA* allele is relatively high among Asians and Africans (4–18%), but low in Hispanics, Europeans, and Japanese populations (<1.5%).

On the EU label, the Special Warnings and Precautions section describes that the HLA-B^*^58:01 allele is associated with the risk of developing allopurinol-related hypersensitivity syndrome and SJS/TEN, and screening for HLA-B^*^58:01 should be considered before initiating treatment in patient subgroups where the prevalence of this allele is known to be high (Table 1). A similar statement is presented in the (Special) Warnings and Precautions section of the Australian and Singaporean labels. The Japanese label shows only the facts from reported papers in the Other Precautions subsection of the Side-Effects section owing to the very low frequency of the variant allele when compared with that observed in Han Chinese. In contrast, no pharmacogenomic description is stated in the US and Canadian labels. The Warnings section of the US drug label states that the drug should be discontinued at the first appearance of skin rash or other signs that may indicate an allergic reaction. A similar description is presented in the Warnings section of the Canadian label.

**Table 1:** Description of pharmacogenomic information in the five drug labels of the United States, European Union (EU) or the United Kingdom (UK), Canada, Australia, Singapore, and Japan.

**Drugs**	**Biomarker**	**US**	**EU or UK**	**Canada**	**Australia**	**Singapore**	**Japan**
Allopurinol	HLA-B^*^58:01	**(Warnings)** • This drug should be discontinued at the first appearance of skin rash or other signs which may indicate an allergic reaction. • In some instances a skin rash may be followed by more severe hypersensitivity reactions such as exfoliative, urticarial, and purpuric lesions, as well as SJS (erythema multiforme exudativum), DRESS and/or generalized vasculitis, irreversible hepatotoxicity, and, on rare occasions, death.	**(EU, Special Warnings and Precautions for Use)** • The HLA-B^*^5801 allele is reportedly associated with the risk of developing allopurinol related hypersensitivity syndrome and SJS/TEN. • Screening for HLA-B^*^5801 should be considered before starting treatment with allopurinol in patient subgroups where the prevalence of this allele is known to be high.	**(Warnings)** • Allopurinol should be discontinued immediately at the appearance of a skin rash, as the rash may be, in some instances, followed by a more severe hypersensitivity reaction, including SJS, DRESS, and TEN.	**(Special Warnings and Precautions for Use)** • The HLA-B^*^5801 allele is known to be associated with the risk of developing allopurinol related hypersensitivity syndrome and SJS/TEN. • Screening for HLA-B^*^5801 should be considered before starting treatment in patient subgroups where the prevalence of this allele is known to be high.	**(Warnings and Precautions)** • The HLA-B^*^5801 allele is reportedly associated with the risk of developing allopurinol related hypersensitivity syndrome and SJS/TEN. • Screening for HLA-B^*^5801 should be considered before starting treatment with allopurinol in patient subgroups where the prevalence of this allele is known to be high.	**(Side Effects/Other Precautions)** • A previous report has presented the association of HLA-B^*^5801 and SJS/TEN in Han Chinese, Japanese and European populations. • Allele frequency of HLA-B^*^5801 in Japanese and European population is very low (0.010–0.020) when compared with that in Han Chinese (0.20–0.30).
Carbamazepine	HLA-B^*^15:02	**(Boxed Warnings)** • HLA-B^*^1502 is found almost exclusively in patients with ancestry across broad areas of Asia. • Patients with ancestry in genetically at-risk populations should be screened for the presence of HLA-B^*^1502 prior to initiating treatment with carbamazepine. • Patients testing positive for the allele should not be treated with carbamazepine unless the benefit clearly outweighs the risk.	**(EU, Special Warnings and Precautions for Use)** • Whenever possible, these individuals should be screened for this allele before starting treatment with carbamazepine or chemically-related active substances. • If patients of these ethnic origins are tested positive for HLA-B^*^1502 allele, the use of eslicarbazepine acetate may be considered if the benefits are thought to exceed risks.	**(Boxed Serious Warnings and Precautions)** • The HLA-B^*^1502 allele is found almost exclusively in individuals with ancestry across broad areas of Asia. • Recommended that physicians consider HLA-B^*^1502 genotyping as a screening tool in genetically at-risk populations. • Until further information is available, the use of carbamazepine and other antiepileptic drugs associated with SJS/TEN should be avoided in patients who test positive for the HLA-B^*^1502 allele.	**(Special Warnings and Precautions for Use)** • Testing for the presence of HLA-B^*^1502 allele should be considered in patients with ancestry in genetically at-risk populations, prior to initiating treatment with carbamazepine. • The use of carbamazepine should be avoided in tested patients who are found to be positive for HLA-B^*^1502 unless the benefits clearly outweigh the risks.	**(Warnings and Precautions)** • Testing for the presence of HLA-B^*^1502 allele is highly recommended prior to the initiation of carbamazepine therapy in new patients of Asian ancestry. • The use of carbamazepine should be avoided in tested patients who are found to be positive for HLA-B^*^1502 unless the benefits clearly outweigh the risks.	**(Side Effects/Other Precautions)** • Citing a paper showing association of HLA-B^*^1502 and SJS/TEN in Han Chinese populations. • Allele frequency of HLA-B^*^1502 in Japanese population is very low (0.001) compared to that in Han Chinese (0.019–0.124)
	HLA-A^*^31:01	**(Warning)** • The risks and benefits of carbamazepine therapy should be weighed before considering carbamazepine in patients known to be positive for HLA-A^*^3101.	**(EU, Special Warnings and Precautions for Use)** • There are insufficient data supporting a recommendation for HLA-A^*^3101 screening before starting carbamazepine or chemically-related compounds treatment. • If patients of European descent or Japanese origin are known to be positive for HLA-A^*^3101 allele, the use of carbamazepine or chemically-related compounds may be considered if the benefits are thought to exceed risks.	**(Boxed Serious Warnings and Precautions)** • Recommending that physicians consider HLA-A^*^3101 genotyping as a screening tool in genetically at-risk populations. • Until further information is available, the use of carbamazepine and other antiepileptic drugs associated with SJS/TEN should be avoided in patients who test positive for the HLA-A^*^3101 allele.	**(Special Warnings and Precautions for Use)** • Testing for the presence of HLA-A^*^3101 allele should be considered in patients with ancestry in genetically at-risk populations, prior to initiating treatment with carbamazepine. • The use of carbamazepine should be avoided in patients who are found to be positive for HLA-A^*^3101, unless the benefits clearly outweigh the risks.	**(Warnings and Precautions)** • Testing for the presence of HLA-A^*^3101 allele should be considered in patients with ancestry in genetically at-risk populations prior to initiating treatment with carbamazepine. • The use of carbamazepine should be avoided in patients who are found to be positive for HLA-A^*^3101, unless the benefits clearly outweigh the risks.	**(Side Effects/Other Precautions)** • Citing a paper showing association of HLA-A^*^3101 and severe cutameous adverse reactions in Japanese. • Allele frequency of HLA-A^*^3101 in Japanese population is relatively high (0.071–0.120).
Oxcarbazepine	HLA-B^*^15:02	**(Warnings)** • Patients who have had hypersensitivity reactions to carbamazepine should be informed that approximately 25–30% patients with reactions are known to experience hypersensitivity reactions with oxcarbazepine. • If signs or symptoms of hypersensitivity develop, oxcarbazepine should be discontinued immediately.	**(UK, Warnings and Precautions)** • The risk of serious skin reactions in patients of Han Chinese or Thai origin associated with carbamazepine or chemically-related compounds may be predicted by testing a blood sample from these patients. • Your doctor should be able to advise if a blood test is necessary before taking oxcarbazepine.	**(Boxed Warnings and Precautions)** • The HLA-B^*^1502 allele is found almost exclusively in individuals with ancestry across broad areas of Asia. • It is therefore, recommended that physicians consider HLA-B^*^1502 genotyping as a screening tool in genetically at-risk populations. • Until further information is available, the use of oxcarbazepine and other anti-epileptic drugs associated with SJS/TEN should be avoided in patients who test positive for the HLA-B^*^1502 allele.	**(Special Warnings and Precautions for Use)** • As the chemical structure of oxcarbazepine is similar to that of carbamazepine, there is a possibility that patients carrying the HLA-B^*^1502 allele also have an increased risk of SJS/TEN skin reactions with oxcarbazepine. • Testing for the presence of the HLA-B^*^1502 allele should be considered in patients with an ancestry of genetically at-risk populations, prior to initiating treatment with oxcarbazepine. • The use of oxcarbazepine should be avoided in tested patients who are found to be positive for HLA-B^*^1502 unless the benefits clearly outweigh the risks.	Not approved	**(Boxed Warnings/Side Effects/Other Precautions)** • Patients treated with oxcarbazepine may present the appearance of severe cutaneous adverse reactions such as TEN, SJS, and DIHS. • A report showing allele frequency of HLA-B^*^1502 and HLA-A^*^3101 in Japanese population.
	HLA-A^*^31:01	• Included in the above section.	• Included in the above section.	**(Boxed Warnings and Precautions)** • Retrospective genome-wide studies in Japanese and Northern European populations reported an association between severe skin reactions (SJS, TEN, DRESS, AGEP, and maculopapular rash) associated with carbamazepine use and the presence of the HLA-A^*^3101 allele in these patients.	**(Special Warnings and Precautions for Use)** • As the chemical structure of oxcarbazepine is similar to that of carbamazepine, there is a possibility that patients carrying the HLA-A^*^3101 allele also have an increased risk of SJS/TEN skin reactions with oxcarbazepine.		• Included in the above section.
Phenytoin	HLA-B^*^15:02	**(Warnings and Precautions)** • HLA-B^*^1502 may be a risk factor for the development of SJS/TEN in patients of Asian ancestry taking other antiepileptic drugs associated with SJS/TEN, including phenytoin. • Consideration should be given to avoiding phenytoin as an alternative for carbamazepine in patients positive for HLA-B^*^1502.	**(UK, Special Warnings and Precautions for Use)** • HLA-B^*^ 1502 may be a risk factor for the development of SJS/TEN in patients of Asian ancestry taking drugs associated with SJS/TEN, including phenytoin. • Consideration should be given to avoiding the use of drugs associated with SJS/TEN, including phenytoin, in HLA-B^*^1502 positive patients when alternative therapies are otherwise equally available.	**(Warnings and Precautions)** • The HLA-B^*^1502 allele is found almost exclusively in individuals with ancestry across broad areas of Asia. • Physicians should consider HLA-B ^*^1502 genotyping as a screening tool in these patients. • The use of phenytoin and other anti-epileptic drugs associated with SJS/TEN should also be avoided in patients who test positive for the HLA-B^*^1502 allele.	**(Special Warnings and Precautions for Use)** • HLA-B^*^1502 may be a risk factor for developing SJS/TEN in patients of Asian ancestry taking drugs associated with SJS/TEN, including phenytoin. • Consideration should be given to avoiding the use of drugs associated with SJS/TEN, including phenytoin, in HLA-B^*^1502-positive patients when alternative therapies are otherwise equally available.	**(Special Warnings and Precautions for Use)** • HLA-B^*^1502 may be a risk factor for the development of SJS/TEN in patients of Asian ancestry taking drugs associated with SJS/TEN, including phenytoin. • Consideration should be given to avoiding the use of drugs associated with SJS/TEN, including phenytoin, in HLA-B^*^1502-positive patients when alternative therapies are otherwise equally available.	**(Side Effects)** • Patients treated with phenytoin may present the appearance of severe cutaneous adverse reactions such as TEN and SJS.
Fosphenytoin	HLA-B^*^15:02	**(Warnings and Precautions)** • Consider avoiding fosphenytoin as an alternative to carbamazepine in patients who are positive for HLA-B^*^1502 or in CYP2C9^*^3 carriers. • Fosphenytoin should be utilized for CYP2C9^*^3 carriers; consider starting at the lower end of the dosage range.	**(UK, Special Warnings and Precautions for Use)** • Fosphenytoin can cause SCARs such as AGEP, exfoliative dermatitis, SJS, TEN and DRESS which can be fatal. • Limited evidence suggests that HLA-B^*^1502 may be a risk factor for the development of SJS/TEN in patients of Asian ancestry taking drugs associated with SJS/TEN, including phenytoin.	**(Warnings)** • The HLA-B^*^1502 allele is found almost exclusively in individuals with ancestry across broad areas of Asia. • Physicians should consider HLA-B ^*^1502 genotyping as a screening tool in these patients. • Until further information is available, the use of fosphenytoin and other anti-epileptic drugs associated with SJS/TEN should also be avoided in patients who test positive for the HLA-B^*^1502 allele.	Not approved	Not approved	**(Side Effects)** • Patients treated with fosphenytoin may present the appearance of severe cutaneous adverse reactions such as TEN and SJS.

*AGEP, acute generalized exanthematous pustulosis; DIHS, drug-induced hypersensitivity syndrome; DRESS, drug reaction with eosinophilia and systemic symptoms; SCAR, severe cutaneous adverse reactions; SJS, Stevens-Johnson syndrome; TEN, toxic epidermal necrolysis*.

The “CPIC Guideline for Allopurinol and HLA-B” recommends that allopurinol is contraindicated for HLA-B^*^58:01 carriers [Table 2; (9, 10)]. Non-carriers of HLA-B^*^58:01 are considered to have a low risk for SCARs. Several clinical non-genetic factors, including renal dysfunction and high-dose allopurinol, have also been associated with the risk of allopurinol hypersensitivity. Furthermore, the CPIC guideline warns that HLA-B^*^58:01 predicts only allopurinol-related SCARs, not other skin reactions such as rash, and this allele marker does not predict the efficacy. Moreover, this guideline recommends that physicians should monitor patients closely, regardless of the genotyping results. Some candidate genetic factors, including HLA-A^*^33:03 and HLA-C^*^03:02, may be associated with allopurinol-related SCARs, but CPIC did not include these factors in the guideline as the strength of evidence was not achieved for inclusion. Importantly, the guideline recommends that allopurinol should not be prescribed to patients who have tested positive for HLA-B^*^58:01. Moreover, patients positive for HLA-B^*^58:01 should be treated with alternative drugs. This guideline suggests that a non-purine xanthine oxidase inhibitor, febuxostat, is available as an alternative to allopurinol hypersensitivity. The CPIC guideline also proposes that the patients' pharmacogenetic information must be incorporated into electronic health records to guide physicians' decisions, including drug selection.

**Table 2:** Recommendations for therapy based on genotype in CPIC guideline.

**Drugs**	**Genotype**	**Therapeutic recommendation**
Allopurinol	HLA-B^*^58:01	Allopurinol is contraindicated.
Carbamazepine Oxcarbazepine	HLA-B^*^15:02 positive	If a patient is carbamazepine- or oxcarbazepine-naïve, do not use both drugs. Optional If a patient has previously used carbamazepine or oxcarbazepine consistently for longer than 3 months without the incidence of cutaneous adverse reactions, cautiously consider the use of carbamazepine or oxcarbazepine.
Carbamazepine	HLA-B^*^15:02 negative and HLA-A^*^31:01 positive	If a patient is carbamazepine-naïve and alternative agents are available, do not use carbamazepine. Optional If the patient is carbamazepine-naïve and alternative agents are unavailable, consider the use of carbamazepine with increased frequency of clinical monitoring. Discontinue therapy at the first evidence of a cutaneous adverse reaction. If a patient has previously used carbamazepine consistently for longer than 3 months without the incidence of cutaneous adverse reactions, cautiously consider the use of carbamazepine.
Phenytoin Fosphenytoin	HLA-B^*^15:02 positive	If the patient is phenytoin naïve, do not use phenytoin/fosphenytoin.
	HLA-B^*^15:02 negative	
	*CYP2C9* intermediate metabolizer (^*^1/^*^3, ^*^2/^*^2)	Consider a 25% reduction of the recommended starting maintenance dose. Subsequent maintenance doses should be adjusted according to therapeutic drug monitoring and response.
	*CYP2C9* poor metabolizer (^*^2/^*^3, ^*^3/^*^3)	Consider a 50% reduction of the recommended starting maintenance dose. Subsequent maintenance doses should be adjusted according to therapeutic drug monitoring and response.

*CPIC, Clinical Pharmacogenetics Implementation Consortium*.

## Carbamazepine and Oxcarbazepine

The antiepileptic carbamazepine is known to occasionally induce SCARs, with a markedly strong association observed between carbamazepine- and oxcarbazepine-induced SJS/TEN and HLA-B^*^15:02 (11–14). HLA-B^*^15:02 is common in East Asians (6.9%), Oceanians (5.4%), and South/Central Asians (4.6%), whereas these are <1% in individuals of other Asians, Caucasians and African Americans. HLA-A^*^31:01 is another risk factor for carbamazepine-related SJS/TEN and other SCARs, including drug reactions with eosinophilia and systemic symptoms (DRESS), and even milder skin reactions such as maculopapular exanthema (MPE). The population frequency of HLA-A^*^31:01 is relatively high in Japanese (8%), South Koreans (5%), and Hispanic/South Americans (6%), but relatively low in South/Central Asians (2%), Caucasians (3%), and African-Americans (1%). In addition to the above two *HLA* alleles, HLA-B^*^57:01 was reported to confer genetic susceptibility to carbamazepine-induced SJS/TEN in Europeans (15).

The Boxed Warnings section of the US label shows that HLA-B^*^15:02 is almost exclusively detected in patients with ancestry across broad areas of Asia (at-risk populations), who should be screened for HLA-B^*^15:02 before initiating treatment. HLA-B^*^15:02-positive patients should not be treated with carbamazepine unless the benefit undoubtedly outweighs the risk. Regarding HLA-A^*^31:01, the Warnings section indicates that positive patients should weigh the risks and benefits before commencing treatment with carbamazepine. On the EU label, the Special Warnings and Precautions section indicate that prescreening for HLA-B^*^15:02, whenever possible, should be performed for populations with a high frequency of this allele, such as Han Chinese and Thai, and carbamazepine should not be prescribed to HLA-B^*^15:02-positive patients. Carbamazepine may be used in HLA-A^*^31:01-positive European and Japanese patients if the benefits outweigh the risks. On the Canadian label, the two *HLA* alleles are described in the Boxed Serious Warnings section presenting recommendations for physicians' consideration of HLA-A^*^31:01 and HLA-B^*^15:02 genotyping as a screening tool in genetically at-risk populations. On the Australian label, the Special Warnings and Precautions section states that prior testing for HLA-A^*^31:01 and HLA-B^*^15:02 alleles should be considered in patients with an ancestry of genetically at-risk populations. On the Singaporean label, testing for the HLA-B^*^15:02 allele is highly recommended before initiation of carbamazepine therapy in new patients of Asian ancestry, whereas testing for the HLA-A^*^31:01 allele should be considered in patients of these at-risk populations, which are described in the Warnings and Precautions section. Only the facts from the reported papers are described in the Japanese label for both alleles in the Other Precautions subsection of the Side Effects section.

Cross-sensitivity has been reported among various antiepileptic drugs and is commonly seen in patients receiving aromatic antiepileptic drugs. Oxcarbazepine is a keto-analog of carbamazepine, with a similar structure, sharing several therapeutic indications and adverse effects with carbamazepine. The HLA-B^*^15:02 allele is strongly associated with a greater risk of SJS and TEN in patients treated with oxcarbazepine (14, 16). The Boxed Warnings and Precautions section of the Canadian label recommend that physicians consider HLA-B^*^15:02 genotyping as a screening tool in genetically at-risk populations. For HLA-A^*^31:01, retrospective genome-wide studies in Japanese and Northern European populations reported an association between severe skin reactions (SJS, TEN, and other SCARs) related to carbamazepine use and the presence of the HLA-A^*^31:01 allele in these patients. The Special Warnings and Precautions section of the Australian label states that prior testing for HLA-B^*^15:02 alleles should be considered in patients presenting an ancestry of genetically at-risk populations. For HLA-A^*^31:01, as the chemical structure of oxcarbazepine is similar to that of carbamazepine, there is a possibility that patients carrying the HLA-A^*^31:01 allele also possess an increased risk of SJS/TEN skin reactions with oxcarbazepine. In contrast, the Warnings section of the US label only states, without description regarding *HLA* alleles, that the drug should be immediately discontinued at the appearance of signs or symptoms of hypersensitivity, as ~25–30% of patients who have had hypersensitivity reactions to carbamazepine will experience hypersensitivity reactions with oxcarbazepine. On the UK label, the Warnings and Precautions section indicates that patients with a risk of serious skin reactions, including Han Chinese and those of Thai origin, should be advised if a blood test is necessary before taking oxcarbazepine. The Boxed Warnings of the Japanese label describes that the drug may result in the appearance of SCARs such as TEN, SJS, and drug-induced hypersensitivity syndrome (DIHS) as the chemical structure of oxcarbazepine is similar to that of carbamazepine.

The CPIC guideline for carbamazepine and oxcarbazepine recommends carbamazepine- or oxcarbazepine-naïve patients who are HLA-A^*^15:02-positive should avoid both drugs owing to the high risk of SJS/TEN unless the benefits outweigh the risk [Table 2; (13, 14)]. Patients without HLA-B^*^15:02 could be prescribed standard therapy according to standard dosing guidelines. For selecting other drugs, limited evidence is available regarding the association between HLA-B^*^15:02 and other aromatic anticonvulsants, and caution is needed when selecting an alternative drug. HLA-B^*^15:02 testing helps to reduce the incidence of carbamazepine- or oxcarbazepine-induced SJS/TEN and select an appropriate treatment. The positive predictive value for carbamazepine-related SJS/TEN was higher than that for oxcarbazepine, and the negative predictive values for both drugs were 100%. Thus, negative test results provide valuable information to determine the use of carbamazepine or oxcarbazepine. HLA-B^*^15:02 is included in HLA-B75 serotypes, with other haplotypes in the HLA-B75 serotype presenting similar structures; moreover, the CPIC guideline states the necessity to consider the potential risk if this information is available. Carbamazepine-naïve patients with HLA-A^*^31:01 should avoid carbamazepine owing to the high risk of SCARs, including SJS/TEN (Table 2). Other anticonvulsants, including lamotrigine, oxcarbazepine, eslicarbazepine, phenytoin, fosphenytoin, and phenobarbital, present limited evidence regarding the association of SCAR onset with HLA-A^*^31:01. Therefore, these drugs are not recommended as alternative drugs in HLA-A^*^31:01. If alternative drugs are unavailable for patients with HLA-A^*^31:01, CPIC guidelines propose considering the use of carbamazepine with a high frequency of clinical monitoring, discontinuing therapy at the first evidence of a cutaneous adverse reaction. Carbamazepine- and oxcarbazepine-related SJS/TEN usually develops within the first 4–28 days of therapy, and patients who have been administering these drugs for more than 3 months are at low risk regardless of HLA-B^*^15:02 and HLA-A^*^31:01 status.

## Phenytoin and Fosphenytoin

Phenytoin is a widely prescribed antiepileptic drug that can cause cutaneous adverse reactions, ranging from a mild rash to SCARs, including DRESS, SJS, and TEN. HLA-B^*^15:02 is associated with phenytoin-related SJS/TEN in Asian populations (17–19). The HLA-B^*^15:02 carrier increased the risk of SJS/TEN, and this association has reported a sensitivity of 36.6% and specificity of 87.2%. Instead, non-carriers of HLA-B^*^15:02 are considered low risk but can potentially develop phenytoin-related SJS/TEN. The frequency of HLA-B^*^15:02 is more common in Oceanic and Asian populations than in European and African populations (see above section). In addition to the *HLA* allele, the genotype of *CYP2C9*, a major metabolizing enzyme for phenytoin, is associated with phenytoin-related SJS/TEN. Some activity-decreasing or no function genetic variants of *CYP2C9*, i.e., ^*^2 and ^*^3, increase the probability of phenytoin-related toxicities. CYP2C9^*^2 is classified as a decreased function allele, and ^*^3 is classified as a no function allele in the CPIC guideline (18, 19). In that, individuals with “one decreased or no function + one function alleles” or “two decreased function alleles” (^*^1/^*^2, ^*^1/^*^3, and ^*^2/^*^2) are classified as intermediate metabolizers, and those with “two no function alleles” or “one no functional + one decreased function alleles (^*^2/^*^3 and ^*^3/^*^3) are classified as poor metabolizers. The frequencies of CYP2C9^*^2 and ^*^3 differ among racial and ethnic groups, commonly observed in European and Hispanic populations (>5%). Fosphenytoin, a prodrug of phenytoin, is mostly metabolized to phenytoin within 2 h.

The Warnings and Precautions section of the US label states that patients positive for HLA-B^*^15:02 should avoid phenytoin and fosphenytoin as an alternative to carbamazepine as HLA-B^*^15:02 may be a risk factor for the development of SJS/TEN in patients of Asian ancestry administering other antiepileptic drugs. In the UK, Canadian, Singaporean, and Australia, a similar description is present on phenytoin labels in the Special Warnings and Precautions section. The Warnings and Precautions section of the Canadian label recommends that physicians consider HLA-B^*^15:02 genotyping as a screening tool. Only the reported facts are described in the Japanese label, stating that the drug may result in the appearance of SCARs such as TEN and SJS. No description for *CYP2C9* alleles was present on drug labels of any country/region. Regarding fosphenytoin, only the UK and Canadian labels describe HLA-B^*^15:02.

The CPIC guideline recommends that an HLA-B^*^15:02 carrier should not use phenytoin and fosphenytoin if the patient is phenytoin-naïve (18, 19). The guideline also recommends the re-initiation of phenytoin with caution in patients who have previously used phenytoin/fosphenytoin for longer than 3 months without the incidence of cutaneous adverse reactions. The CPIC guideline recommends that the phenytoin/fosphenytoin starting dose is reduced by at least 25% for CYP2C9^*^1/^*^3 and ^*^2/^*^2 intermediate metabolizers and 50% for poor metabolizers (^*^2/^*^3 and ^*^3/^*^3), with subsequent maintenance dose adjustment based on therapeutic drug monitoring (Table 2). The guideline further shows the algorithm for the dose based on HLA-B^*^15:02 and *CYP2C9* genotypes, recommending the decision of phenytoin/fosphenytoin use based on the HLA-B^*^15:02 genotype, followed by adjusting the initial dose by the *CYP2C9* genotype. The guideline proposes that patients' pharmacogenetic information must be incorporated into electronic health records to guide physicians' decisions.

## Other Drugs

Sulfone drugs, e.g., dapsone, sulfamethoxazole, and salazosulfapyridine (sulfasalazine), are used for infectious and inflammatory diseases. These drugs sometimes cause hypersensitivity, and HLA alleles have been reported as the risk factors for hypersensitivity in Asian population. HLA-B^*^13:01 was significantly associated with dapsone-related hypersensitivity syndrome in Chinese population (20). HLA-B^*^13:01 was also significantly associated with salazosulfapyridine-induced DRESS in Chinese Han population (21). Co-trimoxazole (CTX), the sulfamethoxazole-trimethoprim combination drug, has been known to cause SCARs, and HLA-B^*^15:02 and HLA-C^*^08:01 were significantly associated with CTX-induced SJS/TEN, and HLA-B^*^13:01 was associated with CTX-induced DRESS in Thai population (22). Moreover, genome-wide association study in CTX hypersensitivity in collaboration of Taiwan, Thai and Malaysia confirmed that HLA-B^*^13:01 was strongly associated with its SCARs (23). Recently, association between HLA^*^11:01 and sulfonamide-related SCAR was shown in Japanese patients (24). Unfortunately, neither the drug labels containing pharmacogenomic information in six investigated countries/region nor CPIC guideline for this drug class has not been released.

Acetaminophen and non-steroidal anti-inflammatory drugs are often included in cold medicine (CM), and CM is known to sometimes induce SJS/TEN. Furthermore, acetaminophen was reported to be significantly related to severe ocular involvements in SJS/TEN patients (25). HLA-A^*^02:06 and HLA-B^*^44:03 were associated with CM-related SJS/TEN with severe ocular complications (SOCs) in Japanese (26). The associations with HLA-A^*^02:06 and HLA-B^*^44:03 were also shown in Korean, and Indian, Brazilian and Thai populations, respectively (27, 28). By meta-analysis, HLA-A^*^02:06, HLA-A^*^33:03, HLA-B^*^44:03, and HLA-C^*^05:01 were significantly associated with CM-induced SJS/TEN with SOCs (29). However, at least for the acetaminophen/paracetamol, no pharmacogenomic information are included in the labels of the six countries/region (although mentioned the risks of SJS/TEN) and no CPIC guideline are released.

## Discussion

Pharmacogenomic information is an important factor for predicting the onset of SJS/TEN in the three discussed drug types. Based on the comparison of drug labels in the US, EU/UK, Canada, Australia, Singapore, and Japan, their description is not well-harmonized, possibly because of differences in the population frequencies of risk alleles, availability of genetic testing, and coverage policies of public/private health insurance. In Japan, genetic testing is not commonly available for SJS/TEN and, in principle, most medical costs are covered by the national health insurance. Although situations tend to vary, CPIC guidelines are a scientifically sound goal for pharmacogenomic implementation. Further and periodical investigations on drug labels and guidelines are important to understand the current worldwide situation, as well as for policy determination regarding the description/utilization of pharmacogenomic information of SJS/TEN in each country.

## Author Contributions

All authors listed have made a substantial, direct and intellectual contribution to the work, and approved it for publication.

## Conflict of Interest

The authors declare that the research was conducted in the absence of any commercial or financial relationships that could be construed as a potential conflict of interest.
